# Optimizing broiler growth performance through balanced net energy, standard ileal digestible lysine, and amylose/amylopectin ratios: a Box-Behnken response surface approach^☆^

**DOI:** 10.1016/j.psj.2025.105287

**Published:** 2025-05-10

**Authors:** Caiwei Luo, Chunxiao Ai, Yao Yu, Jianmin Yuan

**Affiliations:** Department of Animal Nutrition and Feed Science, Key Laboratory of Animal Nutrition and Feeding, College of Animal Science and Technology, China Agricultural University, Beijing 100193, China

**Keywords:** Amino acid, Broiler, Digestive kinetics, Net energy, Starch

## Abstract

This study investigated the effects of net energy (**NE**), standard ileal digestible lysine (**SID Lys**), and amylose/amylopectin (**AM/AP**) ratios on broiler growth performance using a Box-Behnken design. A total of 936 male Arbor Acres Plus broilers (15-35 days post-hatch) were allocated to thirteen treatments with three factors at three levels including NE (2,000, 2,250, 2,500 kcal/kg), SID Lys (1.00 %, 1.20 %, 1.40 %), and AM/AP ratios (0.17, 0.22, 0.27, composed of different ratios of pea starch and waxy corn starch). Growth performance was measured weekly. At the growth stage of 15-20 d, quadratic relationships between dietary NE (*P* = 0.038), SID Lys (*P* = 0.010), AM/AP ratios (*P* = 0.021), and broiler 20 d body weight (**BW**), with optimization occurring at 2,303 kcal/kg NE, 1.24 % SID Lys, and an AM/AP ratio of 0.22. The 15-25 d feed-to-gain ratio (**F/G**) decreased linearly with increasing dietary NE (*P* = 0.038) and SID Lys (*P* = 0.010). At the growth stage of 21-27 d, linear increases in broiler 27 d BW (*P* = 0.007) and 21-27 d body weight gain (**BWG**) (*P* = 0.013) were observed with higher dietary SID Lys levels, reaching a peak at 2,500 kcal/kg NE, 1.40 % SID Lys, and an AM/AP ratio of 0.17. The 21-27 d F/G decreased linearly with increasing dietary NE (*P* < 0.001) and SID Lys (*P* < 0.001) levels. At the 28-35 d growth stage, a significant interaction between NE and SID Lys levels was observed for 35 d BW (*P* = 0.016) and 28-35 d BWG (*P* = 0.007). At 2,500 kcal/kg NE, both 35 d BW and 28-35 d BWG increased with higher SID Lys, whereas at 2,000 kcal/kg NE, they initially increased and then decreased as SID Lys levels rose. There was a significant interaction effect of NE and AM/AP ratio on broiler 28-35 d BWG (*P* = 0.017). Further quadratic curve fitting of 28-35 d BWG and 15-35 d BWG against dietary SID Lys/NE ratio revealed that 28-35 d BWG and 15-35 d BWG were optimized at dietary SID Lys/NE of 5.68 and 5.80 mg/kcal, respectively. These data indicate balancing dietary NE and SID Lys can optimize broiler growth, while lowering the dietary AM/AP ratio further enhances growth performance, likely due to improved starch digestibility and energy release dynamics. This study provides actionable insights for precision nutrition strategies in broiler production.

## Introduction

Maximizing feed utilization efficiency is a key goal in poultry production, which can be achieved through precise nutrition. Compared with the metabolizable energy (**ME**) system, the net energy (**NE**) system offers a more accurate prediction to the energy available for maintenance and growth by accounting for energy losses as heat increment (**HI**) during digestion and metabolism (Barzegar et al., 2019). This enhanced precision contributes to improved feed efficiency and more effective diet formulations. Carré et al. (2014) showed that NE to apparent metabolizable energy (**AME**) ratios of crude protein (**CP**), starch, lipids, and fermentable sugars were 0.760, 0.806, 0.862, and 0.602 in broilers, respectively. Similarly, Wu et al. (2019) found that NE to N-corrected apparent metabolizable energy (**AMEn**) ratios of corn, soy oil, and soybean meal were 0.806, 0.888, and 0.734 in growing broilers, respectively. These findings underscored that the NE system, by considering both nutrient digestibility and energy losses, enables more accurate energy evaluation compared with the AME system, supporting its adoption in broiler diet formulations.

CP and Amino acids (**AAs**) are essential for muscle protein synthesis. As the second limiting AA in corn-soybean meal diets for broilers, lysine (**Lys**) plays a critical role in growth, development, and muscle protein deposition (Sterling et al., 2006; El-Bahr et al., 2021; Luo et al., 2023). As the reference AA in the AA balance model, Lys requirements are best determined based on standard ileal digestibility (**SID**), which accounts for endogenous losses and reflects true AA availability for metabolism. Maintaining optimal SID Lys levels supports broiler growth and development (An et al., 2023), whereas excessive SID Lys may disrupt the metabolic balance, leading to reduced protein utilization efficiency and impaired growth performance (unpublished data). With modern broilers targeting improved body weight gain (**BWG**), feed-to-gain ratio (**F/G**), and breast muscle yield, the balance between dietary energy intake and AA levels is crucial (An et al., 2023; Luo et al., 2023; Yu et al., 2024), especially in low-protein diets. Therefore, a precise SID Lys/NE ratio is key to maximizing growth performance while minimizing production costs.

Research has shown that the SID Lys requirement of broilers was affected by dietary amylose to amylopectin (**AM/AP**) ratios (Yin et al., 2019; Luo et al., 2023; Luo et al., 2024; Luo et al., 2025). With the rising prices and potential supply shortages of corn and soybean meal, field peas present a promising alternative in animal feeds due to their high starch and CP content (Igbasan and Guenter, 1996; Sharma et al., 2021). However, field pea starch primarily comprises c-type AP with a high AM/AP ratio, which can impact nutrient absorption and utilization. In addition, field peas contain anti-nutritional factors (**ANFs**) such as trypsin inhibitors, lectins, and tannins (McNeill et al., 2004). These ANFs may lead to reduced starch and protein digestibility and impaired glucose and AA absorption in the diet.

The synchronized absorption of glucose and AAs in the intestine promotes muscle protein deposition and enhances broiler growth performance (Weurding et al., 2003; Selle and Liu, 2019), as glucose facilitates AA uptake for muscle protein synthesis. Previous studies have demonstrated that AP-rich diets lead to rapid starch digestion and glucose release, providing the energy needed for livestock growth (Zhou et al., 2021; Li et al., 2024; Luo et al., 2024; Luo et al., 2025). Conversely, diets high in AM result in slower digestion, offering sustained energy release, which can benefit livestock growth under nutrient-limited conditions (Weurding et al., 2003; Mahmood et al., 2024). However, the interactive effects among dietary NE levels, SID Lys levels, and AM/AP ratios on broiler growth performance remain poorly understood. Current research typically focuses on these factors individually, overlooking their potential synergistic or antagonistic interactions. Given that dietary energy density may influence AA requirements and that the starch structure regulates glucose release kinetics and subsequent AA utilization efficiency and protein deposition, elucidating the combined effects of NE, SID Lys, and AM/AP ratio is critical for optimizing feed formulations.

We hypothesized that dietary NE levels, SID Lys levels, and AM/AP ratios would interactively influence broiler growth performance. Specifically, we expected that increasing NE would improve feed efficiency, optimal SID Lys levels would maximize muscle protein deposition, and a lower AM/AP ratio would enhance glucose release and AA utilization. To test this hypothesis, a Box-Behnken design (**BBD**) was employed, which provides a robust statistical method for assessing the effects of multiple variables and their interactions on response outcomes (Sharma et al., 2018; Macelline et al., 2021; Macelline et al., 2022). This approach enabled us to systematically evaluate the combined effects of NE, SID Lys, and AM/AP ratios on broiler growth performance, thereby contributing to the development of more precise and effective feeding strategies in broiler production.

## Materials and methods

### Ethics statement

The trial was conducted at the Zhuozhou Poultry Nutrition Research Base of China Agricultural University (Hebei, China). All animal procedures adhered to the Beijing Regulations of Laboratory Animals (Beijing, China) and were approved by the Laboratory Animal Ethical Committee of China Agricultural University (approval number: AW40703202-1-5).

### Trial design and diet formulation

The study utilized a BBD within the Response Surface Methodology (**RSM**) framework to investigate the effects of dietary AME levels (3,050 kcal/kg, 3,175 kcal/kg, and 3,300 kcal/kg), SID Lys levels (1.00 %, 1.20 %, and 1.40 %), and AM/AP ratios (0.17, 0.24, and 0.33) on broiler growth performance. The design strategically selected 13 treatments (Table 1), comprising 12 combinations where two factors varied between their extremes while the third remained at the intermediate level, plus one central point (all factors at medium levels). By avoiding extreme high-low combinations of all three factors simultaneously, the BBD prioritizes biological plausibility and operational feasibility while retaining statistical power to model quadratic effects and two-factor interactions.

**Table 1:** Trial treatment group.

Item	AME, kcal/kg	SID Lys, %	AM/AP
1	3,050	1.00	0.24
2	3,050	1.20	0.17
3	3,050	1.20	0.33
4	3,050	1.40	0.24
5	3,175	1.00	0.17
6	3,175	1.00	0.33
7	3,175	1.20	0.24
8	3,175	1.40	0.17
9	3,175	1.40	0.33
10	3,300	1.00	0.24
11	3,300	1.20	0.17
12	3,300	1.20	0.33
13	3,300	1.40	0.24

Abbreviation: AME = apparent metabolizable energy; SID Lys = standard ileal digestible Lysine; AM = amylose; AP = amylopectin.

936 14-day male *Arbor Acres Plus* broilers were randomly divided into 13 treatment groups with 6 replicates and 12 chickens each. The experimental period was from 15 to 35 days of age. Before diet formulation, the various raw ingredients were mashed separately, mixed, and sampled. Then, their approximate nutrient analysis values and AA contents were detected using near-infrared spectroscopy and referred to the SID AA parameter for broilers (China Feed Database, 2010 version) to calculate the SID AA of the ingredients. The experimental diets were formulated according to the measured results of these ingredients (Table 2). The design values of AME were calculated based on standard ingredient compositions, but slight deviations occurred due to natural variability in the actual chemical composition and energy content of feed ingredients, such as corn, soybean meal, and starch sources. For consistency with the BBD, all modeling and response surface analyses were based on the design values of NE, SID Lys, and AM/AP levels.

**Table 2:** Ingredient and nutrient composition of experiment diets (%, as-fed basis).

Ingredient	1	2	3	4	5	6	7	8	9	10	11	12	13
Corn	46.00	47.00	47.00	40.00	42.60	42.60	43.60	38.00	38.00	39.00	40.00	40.00	41.40
Soybean meal	32.40	30.80	30.80	29.90	33.00	33.00	31.43	30.32	30.32	33.70	32.05	32.05	29.60
Waxy corn starch	6.00	12.00	-	6.00	12.00	-	6.00	12.00	-	6.00	12.00	-	6.00
Pea starch	6.00	-	12.00	6.00	-	12.00	6.00	-	12.00	6.00	-	12.00	6.00
Corn gluten meal	5.00	5.00	5.00	5.00	5.00	5.00	5.00	5.00	5.00	5.00	5.00	5.00	5.00
Soybean oil	0.36	0.13	0.13	2.50	3.20	3.20	2.92	4.80	4.80	6.00	5.76	5.76	5.44
Dicalcium phosphate	1.67	1.68	1.68	1.70	1.67	1.67	1.68	1.70	1.70	1.66	1.69	1.69	1.70
Limestone	1.16	1.18	1.18	0.94	1.16	1.16	1.17	0.98	0.98	1.15	1.15	1.15	1.17
Sodium chloride	0.30	0.30	0.30	0.30	0.30	0.30	0.30	0.30	0.30	0.30	0.30	0.30	0.30
Mineral premix^1^	0.20	0.20	0.20	0.20	0.20	0.20	0.20	0.20	0.20	0.20	0.20	0.20	0.20
Choline chloride (50 %)	0.10	0.10	0.10	0.10	0.10	0.10	0.10	0.10	0.10	0.10	0.10	0.10	0.10
Vitamins premix^2^	0.03	0.03	0.03	0.03	0.03	0.03	0.03	0.03	0.03	0.03	0.03	0.03	0.03
Phytase,10000U/g	0.02	0.02	0.02	0.02	0.02	0.02	0.02	0.02	0.02	0.02	0.02	0.02	0.02
L-Lysine hydrochloride (98 %)	0.13	0.43	0.43	0.73	0.12	0.12	0.42	0.72	0.72	0.11	0.42	0.42	0.74
DL-Methionine (98 %)	0.09	0.26	0.26	0.43	0.10	0.10	0.26	0.43	0.43	0.10	0.27	0.27	0.44
L-Threonine (98.5 %)	-	0.12	0.12	0.29	-	-	0.12	0.29	0.29	-	0.13	0.13	0.29
L-Arginine hydrochloride (98 %)	-	0.14	0.14	0.42	-	-	0.13	0.41	0.41	-	0.14	0.14	0.43
L-Valine (98 %)	-	-	-	0.12	-	-	-	0.12	0.12	-	-	-	0.12
L-Isoleucine (90 %)	-	0.08	0.08	0.25	-	-	0.08	0.25	0.25	-	0.08	0.08	0.25
Zeolite power	0.04	0.02	0.02	4.50	-	-	0.03	3.77	3.77	0.13	0.15	0.15	0.20
L-Tryptophan (98 %)	-	0.01	0.01	0.07	-	-	0.01	0.06	0.06	-	0.01	0.01	0.07
Titanium dioxide	0.50	0.50	0.50	0.50	0.50	0.50	0.50	0.50	0.50	0.50	0.50	0.50	0.50
Total	100	100	100	100	100	100	100	100	100	100	100	100	100
Nutrition level													
AME^3^, kcal/kg	3,050	3,050	3,050	3,050	3,175	3,175	3,175	3,175	3,175	3,300	3,300	3,300	3,300
AME^4^, kcal/kg	3,207	3,209	3,171	3,085	3,329	3,337	3,291	3,156	3,324	3,321	3,499	3,401	3,406
AMEn^4^, kcal/kg	3,190	3,193	3,154	3,069	3,313	3,321	3,276	3,142	3,307	3,286	3,484	3,387	3,391
NE^4^, kcal/kg	1,929	2,153	1,914	1,972	2,189	2,291	2,270	2,090	2,465	2,466	2,528	2,461	2,391
CP^3^, %	21.01	21.01	21.01	20.99	21.00	21.00	21.00	21.00	21.00	21.01	21.01	21.01	20.99
Calcium, %	0.95	0.95	0.95	0.95	0.95	0.95	0.95	0.95	0.95	0.95	0.95	0.95	0.95
NPP, %	0.37	0.37	0.37	0.37	0.37	0.37	0.37	0.37	0.37	0.37	0.37	0.37	0.37
SID Lys^3^, %	1.00	1.20	1.20	1.40	1.00	1.00	1.20	1.40	1.40	1.00	1.21	1.21	1.41
SID Met^3^, %	0.43	0.59	0.59	0.74	0.43	0.43	0.58	0.74	0.74	0.43	0.59	0.59	0.75
SID Met+Cys^3^, %	0.73	0.88	0.88	1.02	0.73	0.73	0.88	1.02	1.02	0.73	0.88	0.88	1.03
SID Thr^3^, %	0.69	0.79	0.79	0.92	0.69	0.69	0.79	0.92	0.92	0.7	0.8	0.8	0.92
SID Val^3^, %	0.91	0.88	0.88	0.95	0.91	0.91	0.88	0.96	0.96	0.91	0.88	0.88	0.95
SID Ile^3^, %	0.77	0.82	0.82	0.95	0.77	0.77	0.82	0.96	0.96	0.77	0.83	0.83	0.95
SID Arg^3^, %	1.19	1.28	1.28	1.50	1.19	1.19	1.28	1.50	1.50	1.20	1.29	1.29	1.51
SID Trp^3^, %	0.20	0.20	0.20	0.250	0.20	0.20	0.20	0.24	0.24	0.20	0.20	0.20	0.25
AM/AP^4^	0.23	0.18	0.28	0.22	0.17	0.27	0.24	0.17	0.27	0.22	0.18	0.27	0.23

Abbreviation: AME = apparent metabolizable energy; AMEn = N-corrected apparent metabolizable energy; NE = net energy; CP = crude protein; NPP = non-phytate phosphorus; AM = amylose; AP = amylopectin.

1 The trace mineral premix provided (per kg of diets) the following: Cu, 16 mg; Zn, 110 mg; Fe, 80 mg; Mn, 120 mg; Se, 0.30 mg; I, 1.50 mg.

2 The vitamin premix provided (per kg of diets) the following: vitamin A, 15,000 IU, vitamin D3, 3,600 IU; vitamin E, 30 IU; vitamin K3, 3.00 mg; vitaminB2, 9.60 mg; vitamin B12, 0.03 mg; biotin, 0.15 mg; folic acid, 1.50 mg; pantothenic acid, 13.80 mg; nicotinic acid, 45 mg.

3 Formulate dietary nutritional levels based on near-infrared spectroscopy measurement results.

4 Actually measured values of nutrient components.

### Bird husbandry

1 day old Arbor Acres Plus male broilers were brought from Beijing Poultry Breeding Co., Ltd (Beijing, China). They were fed a uniform commercial diet from 1 to 14 days of age (AME = 2,920 kcal/kg, CP = 22 %, and SID Lys = 1.18 %). Bird management followed the Arbor Acres Plus broilers guide (Aviagen, 2018). All birds had ad libitum access to pellet feed and water via nipple drinkers. The trial began at 14 days of age, with growth performance measured weekly.

### Growth Performance

On d 20, 27, and 35, after a 10 h fasting period, feed intake (**FI**) and BW of broilers were measured on a cage-by-cage basis. BWG and F/G were then calculated. Mortality was recorded daily.

### Metabolic Assay

The NE of the feed was measured at the Institute of Animal Nutrition and Energy of the Animal Husbandry Branch of the Jilin Academy of Agricultural Sciences (Jilin, Jilin), using its self-developed 12-compartment open-circuit respiratory calorimetry device for poultry. All animal test procedures conformed to the guidelines for animal testing specified by the National Institute of Animal Health of the People's Republic of China. 390 healthy AA male broilers with similar body weights were selected. Each dietary treatment included 6 replicates. Within each replicate, 3 birds were housed in a cage for the determination of AME, while 2 birds were housed in a respiratory calorimetry chamber for the determination of NE. The test period was 7 days, with a pre-feeding period of 4 days (12-15 d) and a formal test period of 3 days (16-18 d). The 3-day testing period was selected to balance the need for accurate metabolic measurements with minimizing stress and maintaining normal physiological conditions in broilers. The AME bioassay was adopted from those described by Bourdillon et al. (1990), and respiratory heat production (**HP**) was measured using indirect calorimetry. To ensure the accuracy of AME measurements, the AME test was performed separately and simultaneously with the NE test. Fasting was used at the beginning and end of the formal trial to ensure the accuracy of correspondence between FI and fecal collection. Oxygen consumption, carbon dioxide production, and gas exchange data were collected continuously in the respiratory calorimetry chamber for 3 days of the formal test period, excluding the feeding and fecal removal time points. Initial BW, final BW, and FI of broilers during the formal trial were recorded.

### Chemical analysis

The feed and feces were crushed and sieved through a size of 40 mesh for routine nutrient determinations. Dry matter was determined by oven drying at 105 °C for 5 h (method 934.01, AOAC 1990). The CP (total nitrogen × 6.25) of feed and feces was determined using the Kjeldahl method (method 954.01, AOAC 1990). The gross energy (**GE**) of feed and feces was determined by oxygen bomb calorimetry based on ISO-1928 using a 6400 automatic isoperibol calorimeter (Parr Instrument Inc., Co., Moline, IL, USA). The bomb calorimeter was calibrated at the beginning of each measurement day using benzoic acid (26.454 MJ/kg) as the standard material to determine the energy equivalent of the system. To ensure the consistency and accuracy of intra-run measurements, a second benzoic acid pellet was combusted at the end of each run for verification. The calibration and validation procedures ensured that the energy recovery remained within acceptable limits throughout the assay period. AM and AP contents in diets were determined according to the instructions of Megazyme commercially available kit by a colorimetric method (code: K-AMYL, Wicklow, Ireland). The formula used in this study is as follows.

HP,kJ=16.18×Oxygenconsumption,L+5.02×Carbondioxideproduction,L

Heatincrement,MJ=Totalheatproduction−Heatproductionbyfasting

$$\text{AME},\;\text{kcal}/\text{kg}=\frac{GE_{\text{diet}}\times \text{FI}-GE_{\text{feces}}\times \text{Fecal}\;\text{output}}{\text{FI}}$$

$$\text{NE},\;\text{kcal}/\text{kg}=\frac{\text{AME}\times \text{FI}-\text{Heat}\;\text{increment}}{\text{FI}}$$

### Statistical analysis

All data were tested for statistically significant outliers according to the Grubbs outlier test. The experimental design, model calculation, and graphical plotting were performed by Design-Expert 13. The significant growth performance indices of the model were selected for plotting the response surface plots (*P* < 0.05), the ternary quadratic regression equations were simulated, and the equations were solved by the partial derivative method to find the optimal combinations of the three factors. Regression models for some of the data (15-20 d and 15-35 d BWG, and FI at all growth stages) did not reach statistical significance (*P* > 0.05) and were therefore excluded from further analysis and graphical interpretation.

## Results

### Analysis of actually measured values of diets

Dietary AM/AP post-measurement gradients were 0.18, 0.23, and 0.28, AMEn post-measurement gradients were 3,150, 3,275, and 3,400 kcal/kg, and NE post-measurement gradients were 2,000, 2,250, and 2,500 kcal/kg (Table 2). All subsequent response surface analyses in this study were conducted using actual measurements of dietary AM/AP and NE as factors.

### Growth performance

The response values of different dietary SID Lys, NE, and AM/AP levels to the growth performance of broilers are shown in Table 3. The above data and Design-Expert 13 software were then used for data analysis. The model statistical fitting results of the response of broiler growth performance to different dietary SID Lys, NE, and AM/AP levels (Table 4) showed significant quadratic curvilinear relationships between 20 d BW (*P* = 0.031), 35 d BW (*P* = 0.019), 28-35 d BWG (*P* = 0.021), and 15-35 d F/G (*P* = 0.002) fitted to different dietary SID Lys, NE, and AM/AP levels. 27 d BW (*P* = 0.017), 21-27 d BWG (*P* = 0.041), 15-20 d F/G (*P* = 0.017), 21-27 d F/G (*P* < 0.001), and 28-35 d F/G (*P* < 0.001) fitted with different dietary SID Lys, NE, and AM/AP levels showed significant linear relationships.

**Table 3:** Experimental response values and model *P*-value of growth performance of broilers to different levels of dietary NE, SID Lys, and AM/AP by using Box-Behnken design.

Item				20 d BW, kg	27 d BW, kg	35 d BW, kg	21-27 d BWG, kg	28-35 d BWG, kg	15-20 d F/G	21-27 d F/G	28-35 d F/G	15-35 d F/G
NE	SID Lys		AM/AP									
2,000	1.00		0.22	0.790	1.309	2.002	0.519	0.714	1.73	1.54	1.64	1.63
2,000	1.20		0.17	0.810	1.309	2.018	0.499	0.708	1.60	1.52	1.56	1.55
2,000	1.20		0.27	0.781	1.269	1.990	0.489	0.721	1.69	1.50	1.51	1.54
2,000	1.40		0.22	0.804	1.327	2.007	0.516	0.680	1.53	1.45	1.54	1.52
2,250	1.00		0.17	0.803	1.287	1.992	0.483	0.697	1.62	1.52	1.58	1.40
2,250	1.00		0.27	0.777	1.258	1.917	0.481	0.659	1.64	1.50	1.61	1.59
2,250	1.20		0.22	0.841	1.346	2.078	0.504	0.732	1.50	1.52	1.52	1.50
2,250	1.40		0.17	0.811	1.351	2.076	0.537	0.725	1.55	1.42	1.43	1.45
2,250	1.40		0.27	0.810	1.328	2.015	0.518	0.688	1.55	1.41	1.48	1.46
2,500	1.00		0.22	0.795	1.296	1.954	0.501	0.658	1.66	1.43	1.58	1.54
2,500	1.20		0.17	0.826	1.332	2.065	0.522	0.732	1.48	1.41	1.44	1.45
2,500	1.20		0.27	0.820	1.341	2.010	0.528	0.669	1.51	1.39	1.53	1.47
2,500	1.40		0.22	0.826	1.348	2.096	0.538	0.730	1.46	1.38	1.41	1.41
Mean				0.807	1.315	2.017	0.510	0.701	1.58	1.46	1.53	1.50
SEM				0.004	0.005	0.009	0.004	0.006	0.016	0.011	0.012	0.008
*P*-value												
Model				0.031	0.017	0.019	0.041	0.021	0.017	< 0.001	< 0.001	0.002
Linear		NE		0.017	0.111	0.072	0.137	0.222	0.038	< 0.001	0.012	< 0.001
		SID Lys		0.015	0.007	0.004	0.013	0.023	0.010	< 0.001	< 0.001	< 0.001
		AM/AP		0.035	0.188	0.012	0.551	0.011	0.344	0.324	0.226	0.161
Cross-product		NE×SID Lys		0.252	-	0.016	-	0.007	-	-	-	0.282
		NE×AM/AP		0.151	-	0.406	-	0.017	-	-	-	0.144
		SID Lys×AM/AP		0.129	-	0.652	-	0.953	-	-	-	0.559
Quadratic		NE^2^		0.038	-	0.106	-	0.132	-	-	-	0.395
		SID Lys^2^		0.010	-	0.020	-	0.015	-	-	-	0.029
		AM/AP^2^		0.021	-	0.030	-	0.075	-	-	-	0.655

It shows that the indicator model is linear with no cross-product or quadratic effects.

Abbreviation: NE = net energy; SID Lys = standard ileal digestible Lysine; AM = amylose; AP = amylopectin; BW = body weight; BWG = body weight gain; F/G = feed to gain ratio; SEM = standard error of means.

**Table 4:** Response surface analysis of the optimal combination of dietary SID Lys, NE, and AM/AP levels on growth performance of broilers.

Item	NE, kcal/kg	SID Lys, %	AM/AP	Optimum response values
20 d BW, kg	2,303	1.24	0.22	0.844
27 d BW, kg	2,490	1.38	0.17	1.360
35 d BW, kg	2,496	1.36	0.19	2.110
21–27 d BWG,kg	2,500	1.40	0.17	0.537
28–35 d BWG,kg	2,465	1.38	0.19	0.746
15–20 d F/G	2,469	1.40	0.17	1.46
21–27 d F/G	2,500	1.40	0.22	1.37
28–35 d F/G	2,488	1.39	0.17	1.41
15–35 d F/G	2,492	1.40	0.18	1.40

Abbreviation: NE = net energy; SID Lys = standard ileal digestible Lysine; AM = amylose; AP = amylopectin; BW = body weight; BWG = body weight gain; F/G = feed to gain ratio.

**Fig. 1:**
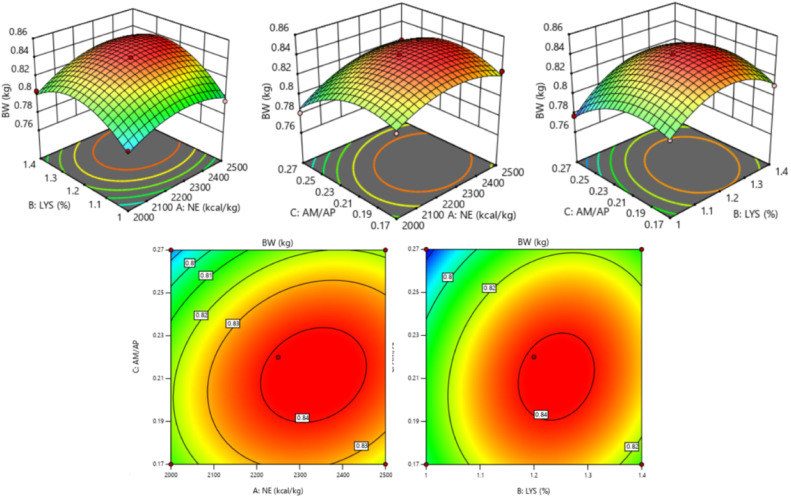
Response surface and contour plots of the relationship between 20 d BW (kg) and SID Lys, NE, and AM/AP. NE = net energy; SID Lys = standard ileal digestible Lysine; AM = amylose; AP = amylopectin; BW = body weight. Fitting equation: 20 d BW = -1.34371+0.001058 NE+0.937611 SID Lys+2.947 AM/AP-2.26×10^−7^ NE^2^-0.342086 SID Lys^2^-7.05 AM/AP², R^2^ = 0.895.

The response of dietary treatments to 20 d BW of broilers is described by the following equation (*R^2^* = 0.895, Fig. 1):

$$20d\text{BW}=-1.34371+0.001058\text{NE}+0.937611\text{SID}\text{Lys}+2.947\text{AM}/\text{AP}-2.26\times 10^{-7}NE^{2}-0.342086\text{SID}\text{Ly}s^{2}-7.05\text{AM}/AP^{2}$$

The response surface and contour plot for 20 d BW is shown in Fig. 1, and there was no interaction between dietary factors (*P* > 0.050). The 20 d BW of broilers showed an increase followed by a decrease as dietary SID Lys (*P* = 0.010), NE (*P* = 0.038), and AM/AP (*P* = 0.021) levels increased. The optimal 20 d BW of 0.844 kg for broilers was achieved when NE was 2,303 kcal/kg, SID Lys was 1.24 %, and AM/AP was 0.22.

**Fig. 2:**
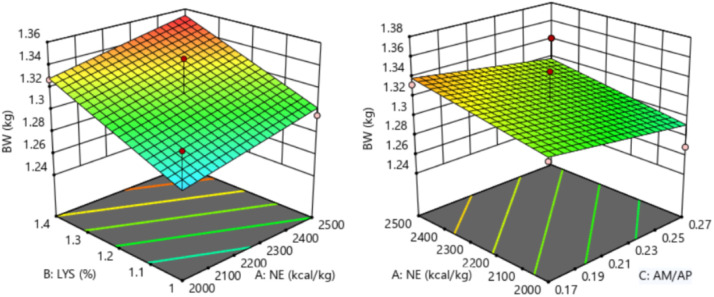
Response surface plots of the relationship between 27 d BW (kg) and SID Lys, NE, and AM/AP. NE = net energy; SID Lys = standard ileal digestible Lysine; AM = amylose; AP = amylopectin; BW = body weight. Fitting equation: 27 d BW=1.11676+0.000051 NE+0.098077 SID Lys-0.2075 AM/AP, R^2^ = 0.546.

The response of dietary treatments to 27 d BW of broilers is described by the following equation (*R^2^* = 0.546, Fig. 2):

27dBW=1.11676+0.000051NE+0.098077SIDLys−0.2075AM/AP

The 27 d BW of broilers showed a linear relationship with the dietary SID Lys levels, which increased with increasing dietary SID Lys levels (*P* = 0.007). The optimal 27 d BW of 1.360 kg for broilers was achieved when NE was 2,490 kcal/kg, SID Lys was 1.38 %, and AM/AP was 0.17.

**Fig. 3:**
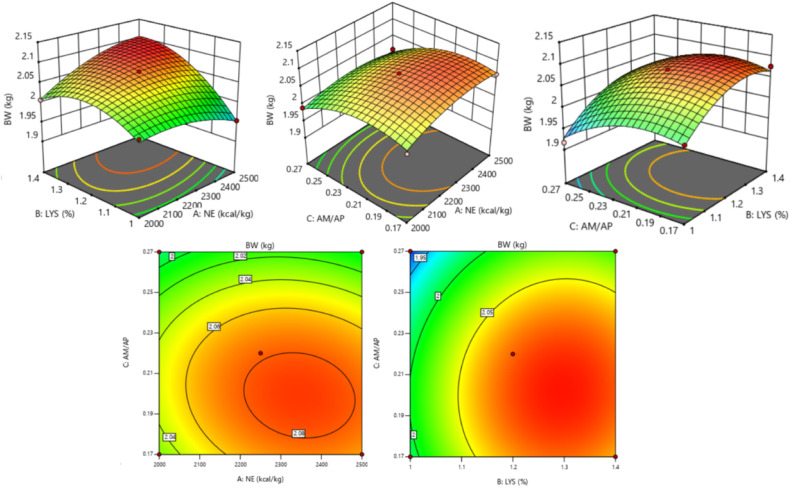
Response surface and contour plots of the relationship between 35 d BW (kg) and SID Lys, NE, and AM/AP. NE = net energy; SID Lys = standard ileal digestible Lysine; AM = amylose; AP = amylopectin; BW = body weight. Fitting equation: 35 d BW = 2.0429-0.000636 NE+0.247438 SID Lys+4.18564 AM/AP+0.000527 NE×SID Lys-0.486581 SID Lys^2^-10.75714 AM/AP^2^, R^2^ = 0.925.

The response of dietary treatments to 35 d BW of broilers is described by the following equation (*R^2^* = 0.925, Fig. 3):

$$35d\text{BW}=2.0429-0.000636\text{NE}+0.247438\text{SID}\text{Lys}+4.18564\text{AM}/\text{AP}+0.000527\text{NE}\times \text{SID}\text{Lys}-0.486581\text{SID}\text{Ly}s^{2}-10.75714\text{AM}/AP^{2}$$

The 35 d BW of broilers increased and then decreased with increasing dietary SID Lys (*P* = 0.020) and AM/AP (*P* = 0.030) levels. There was a significant interaction effect between NE and SID Lys (*P* = 0.016). At the NE level of 2,000 kcal/kg, broiler 35 d BW showed a first increase and then a decrease with increasing SID Lys levels. At the NE level of 2,500 kcal/kg, broiler 35 d BW increased with increasing levels of SID Lys. The optimal 35 d BW of 2.110 kg for broilers was achieved when NE was 2,496 kcal/kg, SID Lys was 1.36 %, and AM/AP was 0.19.

**Fig. 4:**
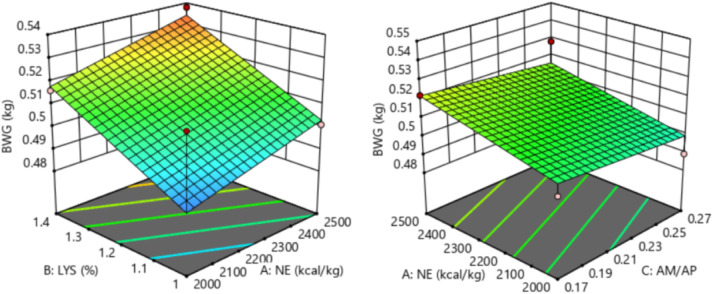
Response surface plots of the relationship between 21-27 d BWG (kg) and SID Lys, NE, and AM/AP. NE = net energy; SID Lys = standard ileal digestible Lysine; AM = amylose; AP = amylopectin; BWG = body weight gain. Fitting equation: 21-27 d BWG = 0.371159+0.000033 NE+0.060096 SID Lys-0.0625 AM/AP, R^2^ = 0.446.

The response of dietary treatments to 21-27 d BWG of broilers is described by the following equation (*R^2^* = 0.446, Fig. 4):

21−27dBWG=0.371159+0.000033NE+0.060096SIDLys−0.0625AM/AP

The 21-27 d BWG of broilers showed a linear relationship with the dietary SID Lys levels (*P* = 0.013), which increased with increasing dietary SID Lys levels. The optimal 21-27 d BWG of 0.537 kg for broilers was achieved when NE was 2,500 kcal/kg, SID Lys was 1.40 %, and AM/AP was 0.17.

**Fig. 5:**
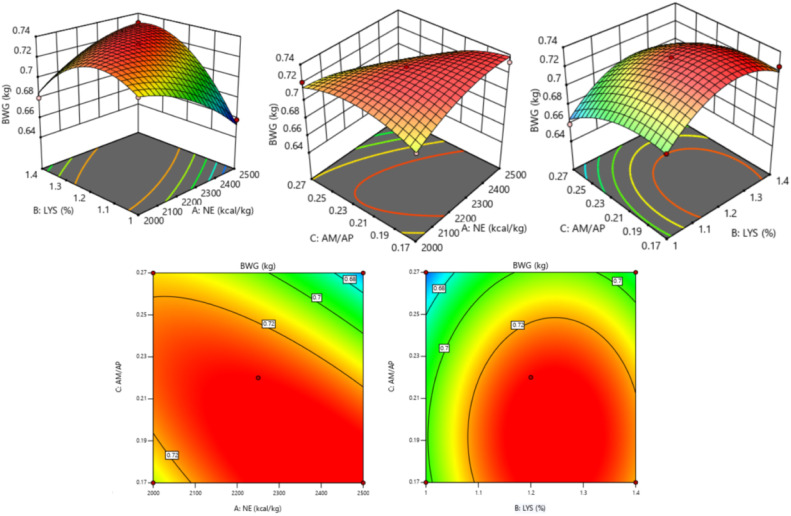
Response surface plots of the relationship between 28-35 d BWG (kg) and SID Lys, NE, and AM/AP. NE = net energy; SID Lys = standard ileal digestible Lysine; AM = amylose; AP = amylopectin; BWG = body weight gain. Fitting equation: 28-35 d BWG = 0.738564-0.000217 NE-0.153654 SID Lys+3.1075 AM/AP+0.000408 NE×SID Lys-0.00152 NE×AM/AP-0.274038 SID Lys^2^, R^2^=0.926.

The response of dietary treatments to 28-35 d BWG of broilers is described by the following equation (*R^2^* = 0.926, Fig. 5):

$$28-35d\text{BWG}=0.738564-0.000217\text{NE}-0.153654\text{SID}\text{Lys}+3.1075\text{AM}/\text{AP}+0.000408\text{NE}\times \text{SID}\text{Lys}-0.00152\text{NE}\times \text{AM}/\text{AP}-0.274038\text{SID}\text{Ly}s^{2}$$

The 28-35 d BWG of broilers increased and then decreased with increasing dietary SID Lys levels (*P* = 0.015) and increased with decreasing AM/AP ratio *(P* = 0.011). There were significant interaction effects between NE and SID Lys (*P* = 0.007) and between NE and AM/AP (*P* = 0.017). At the NE of 2,000 kcal/kg, broiler 28-35 d BWG decreased with increasing SID Lys levels and increased with increasing AM/AP ratio. At the NE of 2,500 kcal/kg, broiler 28-35 d BWG increased with increasing SID Lys levels and decreased with increasing AM/AP ratio.

**Fig. 6:**
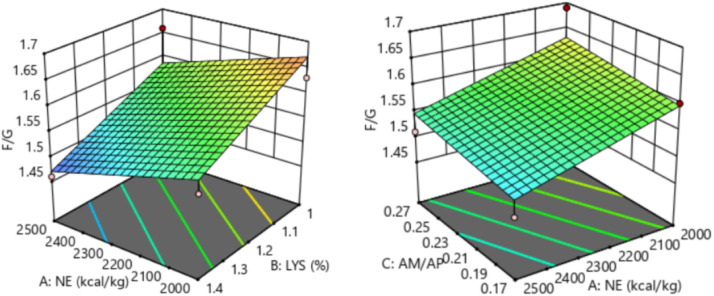
Response surface plots of the relationship between 15-20 d F/G and SID Lys, NE, and AM/AP. NE = net energy; SID Lys = standard ileal digestible Lysine; AM = amylose; AP = amylopectin; F/G = feed to gain ratio. Fitting equation: 15-20 d F/G = 2.16598-0.00017 NE-0.221154 SID Lys+0.35 AM/AP, R^2^ = 0.549.

The response of dietary treatments to 15-20 d F/G of broilers is described by the following equation (*R^2^* = 0.549, Fig. 6):

15−20dF/G=2.16598−0.00017NE−0.221154SIDLys+0.35AM/AP

The 15-20 d F/G of broilers showed a linear relationship with dietary NE and SID Lys levels, which decreased with increasing dietary NE (*P* = 0.038) and SID Lys (*P* = 0.010) levels. The optimal 15-20 d F/G of 1.46 for broilers was achieved when NE was 2,469 kcal/kg, SID Lys was 1.40 %, and AM/AP was 0.17.

**Fig. 7:**
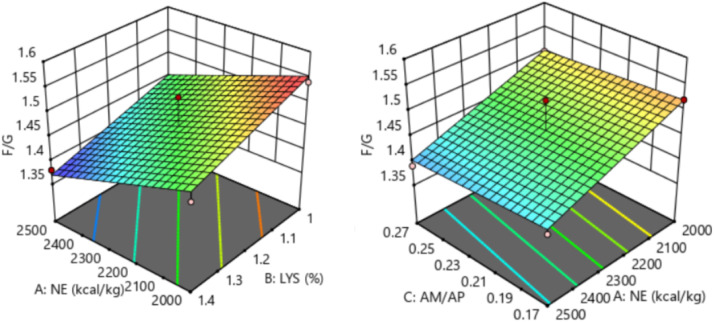
Response surface plots of the relationship between 21-27 d F/G and SID Lys, NE, and AM/AP. NE = net energy; SID Lys = standard ileal digestible Lysine; AM = amylose; AP = amylopectin; F/G = feed to gain ratio. Fitting equation: 21-27 d F/G = 2.15711-0.0002 NE - 0.158654 SID Lys-0.175 AM/AP, R^2^ = 0.828.

The response of dietary treatments to 21-27 d F/G of broilers is described by the following equation (*R^2^* = 0.828, Fig. 7):

21−27dF/G=2.15711−0.0002NE−0.158654SIDLys−0.175AM/AP

The 21-27 d F/G of broilers showed a linear relationship with dietary NE and SID Lys levels, which decreased with increasing dietary NE (*P* < 0.001) and SID Lys (*P* < 0.001) levels. The optimal 21-27 d F/G of 1.37 for broilers was achieved when NE was 2,500 kcal/kg, SID Lys was 1.40 %, and AM/AP was 0.22.

**Fig. 8:**
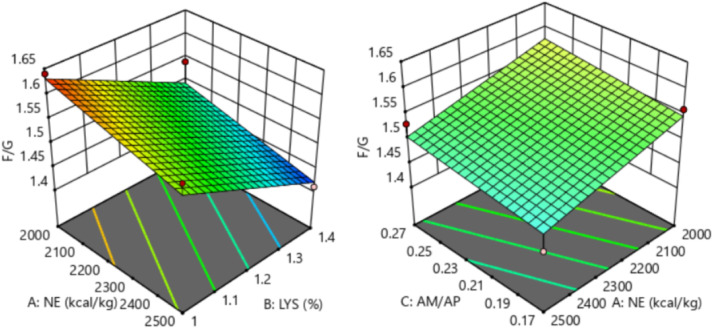
Response surface plots of the relationship between 28-35 d F/G and SID Lys, NE, and AM/AP. NE = net energy; SID Lys = standard ileal digestible Lysine; AM = amylose; AP = amylopectin; F/G = feed to gain ratio. Fitting equation: 28-35 d F/G = 2.13203-0.000145 NE-0.264423 SID Lys+0.3 AM/AP, R^2^ = 0.786.

The response of dietary treatments to 28-35 d F/G of broilers is described by the following equation (*R^2^* = 0.786, Fig. 8):

28−35dF/G=2.13203−0.000145NE−0.264423SIDLys+0.3AM/AP

The 28-35 d F/G of broilers showed a linear relationship with dietary NE and SID Lys levels, which decreased with increasing dietary NE (*P* = 0.012) and SID Lys (*P* < 0.001) levels. The optimal 28-35 d F/G of 1.41 for broilers was achieved when NE was 2,488 kcal/kg, SID Lys was 1.39 %, and AM/AP was 0.17.

**Fig. 9:**
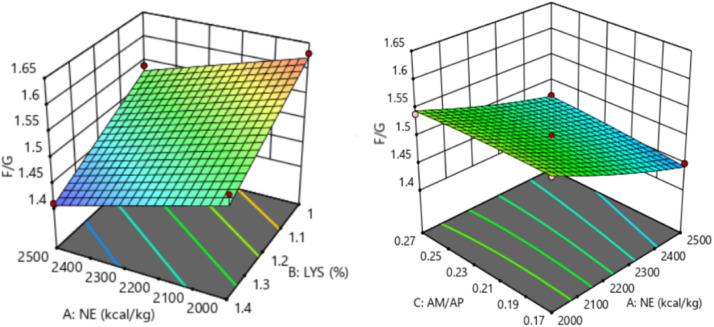
Response surface plots of the relationship between 15-35 d F/G and SID Lys, NE, and AM/AP. NE = net energy; SID Lys = standard ileal digestible Lysine; AM = amylose; AP = amylopectin; F/G = feed to gain ratio. Fitting equation: 15-35 d F/G = 2.69354 - 0.000185 NE - 0.981657 SID Lys+0.1 AM/AP+0.284763 SID Lys^2^, R^2^=0.987.

The response of dietary treatments to 15-35 d F/G of broilers is described by the following equation (*R^2^* = 0.987, Fig. 9):

$$15-35dF/G=2.69354-0.000185\text{NE}-0.981657\text{SID}\text{Lys}+0.1\text{AM}/\text{AP}+0.284763\text{SID}\text{Ly}s^{2}$$

The 15-35 d F/G of broilers showed a linear relationship with dietary NE and SID Lys levels, which decreased with increasing dietary NE (*P* < 0.001) and SID Lys (*P* < 0.001) levels. The optimal 15-35 d F/G of 1.40 for broilers was achieved when NE was 2,492 kcal/kg, SID Lys was 1.40 %, and AM/AP was 0.17.

**Fig. 10:**
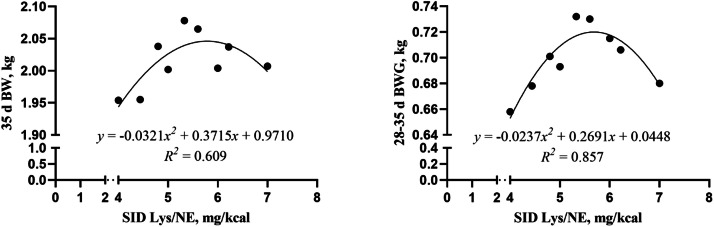
Equation of dietary SID Lys/NE ratios (mg/kcal) with broiler 35 d BW and 28-35 d BWG fitting curve (the growth stage of 28-35 d). NE = net energy; SID Lys = standard ileal digestible Lysine; BW = body weight; BWG = body weight gain.

**Fig. 11:**
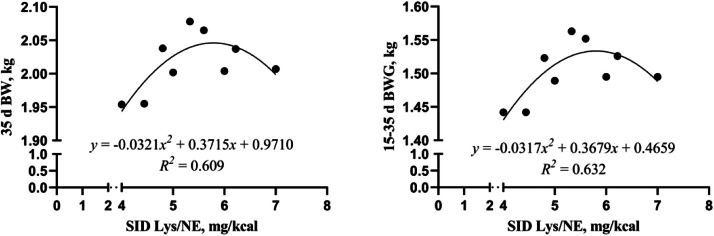
Equation of dietary SID Lys/NE ratios (mg/kcal) with broiler 35 d BW and 15-35 d BWG fitting curve (the growth stage of 15-35 d). NE = net energy; SID Lys = standard ileal digestible Lysine; BW = body weight; BWG = body weight gain.

The present study further fitted the equations of 28-35 d and 15-35 d growth performance of broilers with SID Lys/NE ratio (Fig. 10, Fig. 11) to elucidate more clearly the effect of SID Lys/NE ratio on broiler growth. At the growth stage of 28-35 d, the results of the study revealed a quadratic relationship between the SID Lys/NE ratio and the broiler 35 d BW and 28-35 d BWG. Broiler 35 d BW and 28-35 d BWG were maximized when the SID Lys/NE ratio was 5.79 and 5.68 mg/kcal, respectively.

$$35d\text{BW}:y=-0.0321x^{2}+0.3715x+0.9710,R^{2}=0.609$$

$$28-35d\text{BWG}:y=-0.0237x^{2}+0.2691x+0.0448,R^{2}=0.857$$

At the growth stage of 15-35 d, the results of the study revealed a quadratic relationship between the SID Lys/NE ratio and the broiler 35 d BW and 15-35 d BWG. Broiler 35 d BW and 15-35 d BWG were maximized when the SID Lys/NE ratio was 5.79 and 5.80 mg/kcal, respectively.

$$35d\text{BW}:y=-0.0321x^{2}+0.3715x+0.9710,R^{2}=0.609$$

$$15-35d\text{BWG}:y=-0.0317x^{2}+0.3679x+0.4659,R^{2}=0.632$$

## Discussion

Although the final BW of broilers in the best-performing treatment (Diet 13) did not reach the commercial target proposed by the Arbor Acres Plus Broiler Performance Objectives, this discrepancy may be explained by the use of experimental diets aimed at evaluating nutrient interactions rather than maximizing growth performance. In addition, genetic variation or environmental adaptability may have also contributed. These factors, however, do not diminish the value of the study’s design in systematically evaluating nutrient effects. This study systematically evaluated the linear, quadratic, and interactive effects of NE, SID Lys, and AM/AP ratio on broiler growth performance using a BBD in different growth stages. BBD allowed for efficient modeling with fewer experimental runs while capturing the complex relationships among variables. The analysis identified significant effects and interactions between NE and SID Lys on BW and BWG. The response surface plots and predictive models provide practical insights for optimizing nutrient levels in broiler diets. Broiler growth and development are closely associated with dietary NE and SID Lys levels. The findings of this study demonstrated a significant additive effect between NE and SID Lys levels, particularly concerning 35 d BW (*P* = 0.016) and 28-35 d BWG (*P* = 0.007). Response surface plot analysis indicated that optimal 35 d BW (Fig. 3) and 28-35 d BWG (Fig. 5) were achieved when both NE and SID Lys were increased simultaneously. Further fitting the SID Lys/NE ratio (mg/kcal) to broiler 28-35 d and 15-35d growth performance revealed a quadratic relationship between the above values (Fig. 10, Fig. 11). The optimal SID Lys/NE ratios for achieving maximum 28-35 d BWG and 15-35 d BWG of broilers were 5.68 and 5.80 mg/kcal, respectively. This synergistic effect can be attributed to the role of Lys in promoting protein synthesis (Jin et al., 2019) and NE in supporting energy metabolism (Barzegar et al., 2020). Liu et al. (2019) emphasized the importance of balancing energy and AA supply. Adequate SID Lys/NE ratios are essential to prevent the underutilization of AAs due to energy deficiency. In this study, broilers achieved a peak BW of 2.110 kg at 15-35 d when the NE level was 2496 kcal/kg, the SID Lys level was 1.36 %, and the SID Lys/NE ratio was 5.45 mg/kcal. Sufficient NE ensures adequate energy for growth, while appropriate Lys levels directly contribute to muscle protein deposition. The interaction of these two factors enhances broiler growth performance.

The efficiency of energy utilization in broilers is influenced by the degree of starch digestion (Low et al., 2018). A key finding of this study was the significant interaction between NE and AM/AP ratios on broiler 28-35 d BWG (*P* = 0.017). At an NE level of 2,000 kcal/kg, broiler 28-35 d BWG increased with increasing AM/AP ratio. The AM/AP ratio reflects the rate of starch digestion, with AM-enriched diets having slower digestion rates and providing a more stable and sustained energy release, which may be advantageous under conditions of limited nutrient concentration (Weurding et al., 2003; Mahmood et al., 2024). Yin et al. (2019) reported that nutritional factors regulated AA catabolism in the intestinal mucosa, and dietary AM can reduce AA catabolism, enhancing AA availability in the intestine and improving broiler growth performance. This may explain the enhanced growth performance observed in broilers fed high AM/AP ratio diets (0.27) at lower NE levels (2,000 kcal/kg) in this study. However, at an NE level of 2,500 kcal/kg, broiler 28-35 d BWG increased with decreasing AM/AP ratio. Under sufficient nutrient concentrations, AP-enriched diets are rapidly digested, releasing glucose and stimulating insulin secretion to meet the body`s energy demands (Luo et al., 2023). Studies by Ma et al. (2020) and Gao et al. (2023) indicated that AP-enriched diets increased NE intake and energy retention, enhancing energy utilization and consequently improving the growth performance of livestock, which aligns with the results of this study. These findings suggest that modulating the type of starch at adequate nutrient concentrations can more effectively meet the energy requirements for growth, enabling more efficient energy use for protein synthesis and thereby improving growth efficiency. Although the interaction between SID Lys and AM/AP ratio on broiler growth performance and carcass traits was not significant (*P* > 0.05), its potential effect on protein synthesis should not be overlooked. Lys is a crucial growth-promoting AA for protein synthesis and muscle development. The AM/AP ratio affects the rate of dietary glucose release and energy availability. Numerous studies have indicated that the simultaneous uptake of AAs and glucose during digestion is necessary for maximizing protein synthesis (Weurding et al., 2003; Liu and Selle, 2015; Selle and Liu, 2019; Zhou et al., 2022). An interesting observation in this study was that an AM/AP ratio of 0.18 to 0.21 resulted in optimal performance at high SID Lys levels in both early and late broiler stages. This suggests that broilers have a higher demand for glucose from starch to maintain a synchronized supply of glucose and AA supply in the intestine at high AA levels. This finding contradicts previous results (Weurding et al., 2003; Yin et al., 2019; Mahmood et al., 2024), warranting further research on the interaction between SID Lys and AM/AP ratio.

In the present study, dietary NE, SID Lys, and AM/AP ratio exhibited quadratic or linear relationships with growth performance in addition to the interaction between the factors. It was found that 20 d BW of broilers showed an initial increase followed by a decrease as NE, SID Lys, and AM/AP ratios increased, and 35 d BW of broilers exhibited an initial increase followed by a decline with increasing SID Lys and AM/AP ratios. This quadratic relationship reflects the presence of an optimal level for each of these factors to maximize BW. When nutritional factors are too low or too high, BW gain is hindered due to nutritional deficiencies or metabolic burdens (Herwig et al., 2019; Sharma et al., 2021; Mousa et al., 2023; Yu et al., 2024).

The present study demonstrates that the effects of NE, SID Lys, and AM/AP ratio on broiler growth performance vary across growth stages. During the early phase (15-20 d), NE, SID Lys, and AM/AP all exerted significant linear or quadratic effects on BW. Notably, an AM/AP ratio of 0.22 resulted in optimal BW at this phase, suggesting that a lower AM/AP ratio (rapidly digestible starch) plays a critical role in early gut function and nutrient utilization. The intestinal development of broilers is immature in the early phase; the synchronized release of glucose from rapidly digestible starch with crystalline AAs may enhance insulin secretion patterns, reduce intestinal AA oxidation, and thereby improve protein synthesis and growth performance (Luo et al., 2023; Luo et al., 2024). Moreover, the quadratic effects of both NE and SID Lys on 20 d BW indicate that optimal growth performance during the early phase is highly dependent on balanced energy and AA supply. Excessive or insufficient levels of NE or SID Lys can impair growth performance at this phase. In the grower phase (21-27 d), SID Lys exerted a significant linear influence on both BW and BWG, while NE and AM/AP ratios showed no significant effects. Similar findings were reported by Toghyani et al. (2024), who noted that AA density had a greater impact than ME on BWG during this phase. This likely reflects the sharp increase in protein synthesis, requiring a higher supply of essential AAs, particularly Lys. Additionally, during this phase, BW is maintained through a balance between energy expenditure and FI, both of which are tightly regulated by complex neuroendocrine pathways (Richards and Proszkowiec-Weglarz, 2007), which may attenuate the impact of dietary energy level. During the finisher phase (28-35 d), both SID Lys and AM/AP had significant linear or quadratic effects on BW and BWG, highlighting the importance of dietary starch structure in coordinating energy and AA metabolism during this phase. The glucose release pattern from rapidly digestible starch may better align with high SID Lys availability to support muscle growth and metabolic homeostasis (Luo et al., 2025). In summary, SID Lys serves as a key nutritional regulator throughout the broiler growth cycle, with a lower AM/AP ratio potentially exerting a synergistic effect to enhance performance. These findings underscore the importance of stage-specific precision nutrition strategies that match the dynamic metabolic demands of broilers, thereby optimizing feed efficiency and growth potential.

## Conclusion

This study demonstrated that broilers require stage-specific adjustments in dietary NE, SID Lys, and AM/AP ratios to maximize growth performance. The SID Lys/NE ratios of 5.68 mg/kcal (28-35 d) and 5.80 mg/kcal (15-35 d) maximize BWG and feed efficiency. Lower AM/AP ratios further enhance growth performance, particularly when dietary NE is high. Dynamic nutrient formulations based on growth phases significantly improved the final BW to 2.110 kg and the F/G ratio to 1.40. These findings provide a roadmap for optimizing broiler feed formulations and promoting sustainable poultry production through enhanced energy-AA-starch synergies. Future research should validate these models in commercial settings and explore the molecular mechanisms of starch-AA synchrony in intestinal absorption.

## Declaration of ethics approval

All animal procedures adhered to the Beijing Regulations of Laboratory Animals (Beijing, China) and were approved by the Laboratory Animal Ethical Committee of China Agricultural University (approval number: AW40703202-1-5).

## Funding

This research was supported by National Key R&D Program of China (2021YFD1300404).

## Informed consent statement

Informed consent was obtained from all subjects involved in the study.

## Data availability statement

The data presented in this study are available on request from the corresponding author.

## CRediT authorship contribution statement

**Caiwei Luo:** Conceptualization, Methodology, Investigation, Data curation, Formal analysis, Visualization, Writing – original draft. **Chunxiao Ai:** Conceptualization, Methodology, Investigation, Data curation, Formal analysis. **Yao Yu:** Investigation, Formal analysis, Visualization, Writing – review & editing. **Jianmin Yuan:** Conceptualization, Methodology, Funding acquisition, Resources, Project administration, Supervision, Writing – review & editing.

## Declaration of competing interest

All authors in this study declare that they have no financial or inter-personal conflicts of interest and all sign this statement.

## References

[bib0001] An S.H., Lee B., Choi Y.M., Kong C. (2023). Standardized ileal digestible lysine requirements based on growth performance and histochemical characteristics of male broilers from 10 to 21 d of age. Anim. Nutr..

[bib0002] Aviagen. 2018. Arbor Acres Broiler Management Handbook, Huntsville, Alabama, USA.

[bib0003] Barzegar S., Wu S.B., Choct M., Swick R.A. (2020). Factors affecting energy metabolism and evaluating net energy of poultry feed. Poult. Sci..

[bib0004] Barzegar S., Wu S.B., Noblet J., Choct M., Swick R.A. (2019). Energy efficiency and net energy prediction of feed in laying hens. Poult. Sci..

[bib0005] Bourdillon A., Carré B., Conan L., Duperray J., Huyghebaert G., Leclercq B., Lessire M., McNab J., Wiseman J. (1990). European reference method for the *in vivo* determination of metabolisable energy with adult cockerels: reproducibility, effect of food intake and comparison with individual laboratory methods. Br. Poult. Sci..

[bib0006] Carré B., Lessire M., Juin H. (2014). Prediction of the net energy value of broiler diets. Animal.

[bib0007] El-Bahr S.M., Shousha S., Alfattah M.A., Al-Sultan S., Khattab W., Sabeq I.I., Ahmed-Farid O., El-Garhy O., Albusadah K.A., Alhojaily S., Shehab A. (2021). Enrichment of broiler chickens' Meat with dietary linseed oil and lysine mixtures: influence on nutritional value, Carcass characteristics and oxidative stress biomarkers. Foods.

[bib0008] Gao X., Yu B., Yu J., Mao X., Huang Z., Luo Y., Luo J., Zheng P., Yan H., He J., Chen D. (2023). Effects of different starch structures on energy metabolism in pigs. J. Anim. Sci. Biotechnol..

[bib0009] Herwig E., Abbott D., Schwean-Lardner K.V., Classen H.L. (2019). Effect of rate and extent of starch digestion on broiler chicken performance. Poult. Sci..

[bib0010] Igbasan F.A., Guenter W. (1996). The evaluation and enhancement of the nutritive value of yellow-, green- and brown-seeded pea cultivars for unpelleted diets given to broiler chickens. Anim. Feed Sci. Technol..

[bib0011] Jin C.L., Zhang Z.M., Ye J.L., Gao C.Q., Yan H.C., Li H.C., Yang J.Z., Wang X.Q. (2019). Lysine-induced swine satellite cell migration is mediated by the FAK pathway. Food Funct..

[bib0012] Li Z., Li Y., Zhao Y., Wang G., Liu R., Li Y., Aftab Q., Sun Z., Zhong Q. (2024). Effects of the kinetic pattern of dietary glucose release on nitrogen utilization, the portal amino acid profile, and nutrient transporter expression in intestinal enterocytes in piglets. J. Anim. Sci. Biotechnol..

[bib0013] Liu S.Y., Naranjo V.D., Chrystal P.V., Buyse J., Selle P.H. (2019). Box-Behnken optimisation of growth performance, plasma metabolites and carcass traits as influenced by dietary energy, amino acid and starch to lipid ratios in broiler chickens. PLoS One.

[bib0014] Liu S., Selle P. (2015). A consideration of starch and protein digestive dynamics in chicken-meat production. World's Poult. Sci. J..

[bib0015] Low J.Y.Q., Lacy K.E., McBride R.L., Keast R.S.J. (2018). The associations between oral complex carbohydrate sensitivity, BMI, liking, and consumption of complex carbohydrate based foods. J. Food Sci..

[bib0016] Luo C., Chen Y., Yin D., Yuan J. (2023). Effects of different dietary starch sources and digestible lysine levels on carcass traits, serum metabolites, liver lipid and breast muscle protein metabolism in broiler chickens. Animals.

[bib0017] Luo C., Wang J., Jiang W., Yin D., Meng G., Wang J., Xu J., Yuan J. (2024). Different starch sources and amino acid levels on growth performance, starch and amino acids digestion, absorption and metabolism of 0- to 3-week-old broilers fed low protein diet. Anim. Nutr..

[bib0018] Luo C., Yu Y., Meng G., Yuan J. (2025). Slowly digestible starch impairs growth performance of broiler chickens offered low-protein diet supplemental higher amino acid densities by inhibiting the utilization of intestinal amino acid. J. Anim. Sci. Biotechnol..

[bib0019] Ma J., Yang T., Yang M., Yan Z., Zhao L., Yao L., Chen J., Chen Q., Tan B., Li T., Yin J., Yin Y. (2020). Effects of dietary amylose/amylopectin ratio and amylase on growth performance, energy and starch digestibility, and digestive enzymes in broilers. J. Anim. Physiol. Anim. Nutr..

[bib0020] Macelline S.P., Chrystal P.V., Greenhalgh S., Toghyani M., Selle P.H., Liu S.Y. (2021). Evaluation of dietary crude protein concentrations, fishmeal, and sorghum inclusions in broiler chickens offered wheat-based diet via Box-Behnken response surface design. PLoS One.

[bib0021] Macelline S.P., Chrystal P.V., Selle P.H., Liu S.Y. (2022). Protein sources and starch-protein digestive dynamics manipulate growth performance in broiler chickens defined by an equilateral-triangle response surface design. Anim. Nutr..

[bib0022] Mahmood T., Vieco-Saiz N., Consuegra J., Mercier Y. (2024). Inclusion of slowly digestible starch source is a promising strategy than reducing starch to protein ratio in low protein broiler diets. Poult. Sci..

[bib0023] McNeill L., Bernard K., MacLeod M. (2004). Food intake, growth rate, food conversion and food choice in broilers fed on diets high in rapeseed meal and pea meal, with observations on sensory evaluation of the resulting poultry meat. Brit. Poultry Sci..

[bib0024] Mousa M.A., Asman A.S., Ali R.M.J., Sayed R.K.A., Majrashi K.A., Fakiha K.G., Alhotan R.A., Selim S. (2023). Impacts of dietary lysine and crude protein on performance, hepatic and renal functions, biochemical parameters, and histomorphology of small intestine, liver, and kidney in broiler chickens. Vet. Sci..

[bib0025] Richards M.P., Proszkowiec-Weglarz M. (2007). Mechanisms regulating feed intake, energy expenditure, and body weight in poultry. Poult. Sci..

[bib0026] Selle P.H., Liu S.Y. (2019). The relevance of starch and protein digestive dynamics in poultry. J. Appl. Poultry Res..

[bib0027] Sharma N.K., Ban Z., Classen H.L., Yang H., Yan X., Choct M., Wu S.B. (2021). Net energy, energy utilization, and nitrogen and energy balance affected by dietary pea supplementation in broilers. Anim. Nutr..

[bib0028] Sharma N.K., Choct M., Toghyani M., Laurenson Y., Girish C.K., Swick R.A. (2018). Dietary energy, digestible lysine, and available phosphorus levels affect growth performance, carcass traits, and amino acid digestibility of broilers. Poult. Sci..

[bib0029] Sterling K.G., Pesti G.M., Bakalli R.I. (2006). Performance of different broiler genotypes fed diets with varying levels of dietary crude protein and lysine. Poult. Sci..

[bib0030] Toghyani M., MacElline S., Selle P.H., Liu S.Y. (2024). Interactive effects of dietary energy levels with amino acid density on growth performance and optimal digestible lys to energy ratio of male broiler chickens. Poult. Sci..

[bib0031] Weurding R.E., Enting H., Verstegen M.W. (2003). The relation between starch digestion rate and amino acid level for broiler chickens. Poult. Sci..

[bib0032] Wu S.-B., Swick R.A., Noblet J., Rodgers N., Cadogan D., Choct M. (2019). Net energy prediction and energy efficiency of feed for broiler chickens. Poult. Sci..

[bib0033] Yin D., Selle P.H., Moss A.F., Wang Y., Dong X., Xiao Z., Guo Y., Yuan J. (2019). Influence of starch sources and dietary protein levels on intestinal functionality and intestinal mucosal amino acids catabolism in broiler chickens. J. Anim. Sci. Biotechnol..

[bib0034] Yu Y., Ai C., Luo C., Yuan J. (2024). Effect of dietary crude protein and apparent metabolizable energy levels on growth performance, nitrogen utilization, serum parameter, protein synthesis, and amino acid metabolism of 1- to 10-day-old male broilers. Int. J. Mol. Sci..

[bib0035] Zhou J., Wang L., Yang L., Yang G., Zeng X., Qiao S. (2022). Different dietary starch patterns in low-protein diets: effect on nitrogen efficiency, nutrient metabolism, and intestinal flora in growing pigs. J. Anim. Sci. Biotechnol..

[bib0036] Zhou J., Wang L., Zhou J., Zeng X., Qiao S. (2021). Effects of using cassava as an amylopectin source in low protein diets on growth performance, nitrogen efficiency, and postprandial changes in plasma glucose and related hormones concentrations of growing pigs. J. Anim. Sci..

